# Diagnostic Test Accuracy and Semi-Quantitative Metrics of ^18^F-FDG PET in Assessing Treatment Response in Skull Base Osteomyelitis and Necrotising Otitis Externa: A Systematic Review and Meta-Analysis

**DOI:** 10.3390/tomography12030032

**Published:** 2026-03-02

**Authors:** Mark Laidlaw, Maya Reid, Sukanya Rajiv, Jean-Marc Gerard

**Affiliations:** 1 Department of Otolaryngology, Royal Victorian Eye and Ear Hospital, Melbourne, VIC 3002, Australia; 2 Department of Otolaryngology, Head and Neck Surgery, Canberra Health Service, Canberra, ACT 2605, Australia; 3 Victorian Cochlear Implant Program, Royal Victorian Eye and Ear Hospital, Melbourne, VIC 3002, Australia; 4 Department of Otolaryngology, University of Melbourne, Melbourne, VIC 3010, Australia

**Keywords:** fluorodeoxyglucose F18, positron emission tomography, skull base osteomyelitis, necrotising otitis externa, malignant otitis externa, treatment monitoring, diagnostic test accuracy, systematic review, meta-analysis

## Abstract

Background/Objectives: Skull base osteomyelitis and necrotising otitis externa require prolonged antibiotic therapy, yet determining optimal treatment cessation timing remains challenging. Conventional imaging modalities demonstrate persistent abnormalities beyond infection resolution, confounding treatment decisions. This systematic review evaluated the diagnostic test accuracy of ^18^F-fluorodeoxyglucose positron emission tomography (^18^F-FDG PET) for treatment response monitoring in skull base osteomyelitis and necrotising otitis externa. Methods: We conducted a systematic review following PRISMA-DTA guidelines, searching MEDLINE, Embase, CENTRAL, CINAHL, Scopus, and Web of Science from inception to November 2025. Studies evaluating ^18^F-FDG PET diagnostic accuracy for treatment response assessment in confirmed skull base osteomyelitis or necrotising otitis externa were included. Two reviewers independently screened studies, extracted data, and assessed risk of bias using QUADAS-2. Bivariate random-effects meta-analysis was performed using MetaBayesDTA to obtain pooled sensitivity and specificity. Results: Eight studies comprising 154 lesions contributed to the primary analysis. Pooled sensitivity was 95.2% (95% credible interval 85.6–99.0%) and pooled specificity was 89.1% (95% credible interval 70.7–96.7%). The positive likelihood ratio was 8.7 (95% credible interval 3.2–28.4) and negative likelihood ratio was 0.05 (95% credible interval 0.01–0.17), with a diagnostic odds ratio of 172.0. Seven studies evaluating detection rate at initial presentation yielded a pooled rate of 96.1% (95% confidence interval 91.3–98.3%). SUVmax was the most frequently used metabolic parameter. Conclusions: ^18^F-FDG PET, specifically using SUVmax, demonstrates high sensitivity and good specificity for treatment response monitoring, with excellent capacity to rule out persistent infection. However, evidence quality is limited by retrospective designs and substantial heterogeneity. Prospective studies with standardised thresholds are needed to validate clinical utility.

## Simple Summary

Skull base osteomyelitis is a severe bone infection near the ear requiring prolonged antibiotic treatment. Clinicians face difficulty determining when to safely stop antibiotics, as stopping too early risks dangerous relapse, while continuing too long causes medication side effects. Traditional imaging cannot distinguish healing bone from active infection. This study analysed whether PET scans, which detect metabolic activity, can accurately identify persistent infection during treatment. Results showed PET scans effectively ruled out active infection with 95% sensitivity and 89% specificity. These findings suggest PET imaging could help clinicians make safer decisions about when to discontinue antibiotics, though larger prospective studies are needed to establish standardised guidelines.

## 1. Introduction

Skull base osteomyelitis (SBO) is an uncommon but potentially devastating infection. Necrotising otitis externa (NOE), the contemporary term for malignant otitis externa (MOE), represents a subset of SBO characterised by temporal bone osteomyelitis secondary to external auditory canal infection [1,2]. Despite its low estimated prevalence of 0.2 to 1.2 cases per 100,000 patients, the incidence is increasing. The condition carries mortality rates of 2.5% to 19% and treatment failure and relapse rates of 22% and 7%, respectively [3,4,5,6].

Patients typically present with intractable otalgia, particularly in elderly, immunocompromised, or diabetic individuals. Diabetes is present in approximately 80% of cases [6,7,8]. Whilst Pseudomonas Aeruginosa remains most common (62–65% of cases), culture-negative cases are increasingly recognised [8,9]. Diagnosis requires clinical suspicion supported by imaging confirmation of bony erosion [10,11]. Treatment has evolved from surgery to prolonged antibiotics [12,13,14,15,16,17].

A critical challenge is determining optimal treatment cessation time. Premature discontinuation risks devastating relapse, whilst unnecessarily prolonged therapy predisposes to antibiotic toxicity, risks antimicrobial resistance, and results in increased healthcare costs. Conventional imaging demonstrates significant limitations. Computed tomography (CT) shows late and slow-to-resolve changes [11,18,19,20]; magnetic resonance imaging (MRI) findings are also slow to resolve with clinical improvement [21]; technetium (Tc-99m) bone scintigraphy and Gallium (Ga-67) scans similarly remain positive due to bony remodelling [22,23,24]. These modalities show persistent abnormalities extending beyond active disease, confounding the determination of ongoing infection versus post-infectious changes. Furthermore, a meta-analysis of the diagnostic accuracy of Tc-99m bone scintigraphy and Ga-67 found that pooled sensitivities were 85.1% (95% CI, 72.0–98.1%) and 71.2% (95% CI, 55.1–87.3%), respectively, and that neither modality was effective in assessment of resolution [25].

Fluorine-18-fluorodeoxyglucose positron emission tomography (^18^F-FDG PET) offers an alternative approach. Its mechanism of localisation is based on glucose metabolism in activated inflammatory cells mediated through upregulated GLUT transporters [26,27]. Integration with CT or MRI combines metabolic and anatomical information. PET is widely available due to its oncological applications and demonstrates 96% sensitivity and 91% specificity for chronic osteomyelitis [24]. Evidence supports PET utility in osteomyelitis at other sites, including diabetic foot infections and other forms of osteomyelitis [24,28,29,30], but evidence is still emerging regarding its use in diagnosing and guiding management in skull base osteomyelitis. Disease-specific, standardised PET response criteria and consensus guideline recommendations for PET-guided treatment endpoints in SBO/NOE remain limited. Consequently, interest in ^18^F-FDG PET for response assessment in SBO/NOE has grown largely by extrapolation from its established utility in chronic osteomyelitis at other anatomical sites, but the skull base evidence base remains recent and heterogeneous.

No definitive cutoff values for safe treatment cessation exist for ^18^F-FDG PET in SBO and NOE, and no systematic review has synthesised evidence regarding diagnostic test accuracy of ^18^F-FDG PET for treatment response monitoring in SBO and NOE. A synthesis of the emerging SBO/NOE-specific evidence is therefore needed to inform clinical decision-making and support future development of standardised response criteria.

### Objectives

This systematic review and meta-analysis aims to evaluate the diagnostic test accuracy of ^18^F-fluorodeoxyglucose positron emission tomography (^18^F-FDG PET), either standalone or combined with computed tomography (CT) or magnetic resonance imaging (MRI), in patients with skull base osteomyelitis (SBO), including necrotising (malignant) otitis externa (NOE/MOE).

## 2. Methods

### 2.1. Review Objectives

The review objectives of this systematic review are as follows:

#### 2.1.1. Primary Objective

To determine the diagnostic test accuracy (sensitivity and specificity) of ^18^F-FDG PET for assessment of treatment response, including detection of persistent or recurrent active infection, in patients with confirmed SBO/MOE undergoing antimicrobial therapy.

#### 2.1.2. Secondary Objectives

There were two secondary objectives:

1. To determine the detection rate of ^18^F-FDG PET in patients with confirmed SBO/MOE at the time of initial imaging.
2. To evaluate semi-quantitative PET measures and imaging thresholds used to classify ^18^F-FDG PET studies as positive or negative for SBO/MOE.

Secondary Objective 1 represents an exploratory deviation from the registered protocol. The literature search identified that almost all included studies enrolled patients with confirmed rather than suspected SBO/NOE, with the exception of one study [31], precluding meta-analysis of diagnostic test accuracy at the point of initial clinical suspicion as originally planned. Instead, we evaluated the proportion of positive ^18^F-FDG PET findings in patients with disease confirmed by other reference standards, providing insight into the detection rate rather than true diagnostic accuracy (sensitivity and specificity) at initial presentation.

### 2.2. Protocol and Registration

This systematic review was conducted in accordance with the Preferred Reporting Items for Systematic Review and Meta-Analyses of Diagnostic Test Accuracy (PRISMA-DTA) guidelines [32] and the Cochrane Handbook for Systematic Reviews of Diagnostic Test Accuracy [33]. The review protocol was prospectively registered in the PROSPERO database (registration number: CRD420251244918).

### 2.3. Search

We searched the following electronic databases from database inception to 26 November 2025: MEDLINE, Embase, Cochrane Central Register of Controlled Trials (CENTRAL), Cochrane Library, CINAHL, Scopus, and Web of Science. Only English-language studies were eligible for inclusion. Citation searching of included studies was performed using Web of Science. Only English studies were evaluated, but no date limitations were applied.

### 2.4. Study Selection and Data Collection

One reviewer independently screened titles and abstracts. Two reviewers (ML and MR) then independently reviewed full texts of potentially eligible studies, with discrepancies resolved by discussion or by a third reviewer when needed. This systematic review used Covidence (Veritas Health Innovation, Melbourne, Australia) to manage the literature screening process [34]. Data were extracted independently by two reviewers (ML and MR) using a standardised data extraction form. Discrepancies were resolved through discussion or, when necessary, by a third reviewer (SR).

### 2.5. Inclusion Criteria

Inclusion criteria were defined according to the population, index test, comparator, and outcomes (PICO) framework.

- Population: Patients of any age with suspected or confirmed skull base osteomyelitis (SBO), including otogenic SBO/necrotising (malignant) otitis externa (NOE/MOE) and central SBO, undergoing imaging for assessment of treatment response (and/or initial evaluation where data permitted).
- Index test: 18F-FDG PET (standalone PET, PET/CT, or PET/MRI) using qualitative or semi-quantitative assessment methods (visual grading, standardised uptake value thresholds, response categories).
- Comparator: Composite reference standards, including clinical diagnosis, microbiological and/or histopathological results, non-PET imaging, or prespecified combinations of these elements, acknowledging the absence of a single perfect reference standard for SBO/MOE and reflecting real-world clinical practice.
- Outcomes: Per-lesion sensitivity and specificity for treatment response assessment (primary outcome); detection rate at initial imaging (secondary outcome); semi-quantitative imaging parameters where reported.

### 2.6. Exclusion Criteria

Studies were excluded if they met the following exclusion criteria:

- Population:
  - Uncomplicated otitis externa or isolated middle ear/mastoid infection without skull base involvement;
  - Other osteomyelitis sites (long bone, vertebral, diabetic foot) without a distinct skull base cohort;
  - Mixed head and neck cohorts where SBO/MOE data could not be separated.
- Index test:
  - PET studies using non-FDG tracers only (e.g., ^67^Ga);
  - Imaging without PET component (standalone CT, MRI, SPECT, planar scintigraphy);
  - ^18^F-FDG-labelled autologous leukocyte PET (distinct index test with different preparation/imaging characteristics);
  - Studies from which per-lesion diagnostic accuracy data could not be extracted or derived.
- Study design:
  - Case reports, narrative reviews, editorials, conference abstracts without full-text availability;
  - Non-English language publications.

### 2.7. Data Extraction of Diagnostic Accuracy Measures

The principal diagnostic accuracy measures were sensitivity and specificity, calculated on a per-lesion basis. The unit of assessment was per-lesion rather than per-patient because some studies included patients with bilateral skull base involvement or patients who developed contralateral disease at a later point, with each instance treated as a separate case. Most included studies reported unilateral disease. Where bilateral or contralateral involvement was noted, lesion-level outcomes were not consistently reported in a way that allowed linkage of multiple lesions to individual patients; therefore, analysis at the patient level was not possible from published data. We acknowledge that the bivariate model assumes independent observations and that any within-patient correlation could lead to underestimation of uncertainty.

For each included study, we extracted or calculated true-positive, false-positive, false-negative, and true-negative values to construct 2 × 2 contingency tables. From these data, we derived sensitivity, specificity, positive and negative likelihood ratios, and diagnostic odds ratios with 95% confidence intervals where appropriate.

For the secondary outcome of detection rate at initial presentation, we calculated the proportion of true positives among all lesions with confirmed disease. This metric differs from true sensitivity because most studies enrolled only patients with already-confirmed disease by the reference standard, without disease-free controls, thereby lacking false-positive and true-negative data necessary for conventional diagnostic accuracy calculations.

### 2.8. Statistical Synthesis Methods

#### 2.8.1. Primary Outcome (Treatment Response Assessment)

The diagnostic test meta-analysis was conducted using the MetaBayesDTA online tool [35,36,37]. Where at least four studies contributed data [38], we used bivariate meta-analysis implemented in MetaBayesDTA to obtain pooled sensitivity and specificity with 95% credible intervals, summary receiver operating characteristic (SROC) curves, and diagnostic odds ratios. Random-effects models were prespecified for all meta-analyses, as our aim was to estimate average effects across studies of various design, patient demographic characteristics and study setting [39].

#### 2.8.2. Secondary Outcome (Diagnostic Detection Proportion)

R statistical software (version 4.5.2) and the meta 8.2-1 package were employed for the weighted pooled univariate meta-analysis of PET detection at the point of diagnosis [40,41]. We calculated the detection rate as the proportion of true positives among all cases with confirmed disease. This post hoc approach was necessary because all studies except one exclusively enrolled patients with already-confirmed disease by the reference standard, precluding calculation of true sensitivity and specificity, which require a disease-free comparator group. We calculated pooled proportions for the detection rate using a random-effects model. Between-study heterogeneity was quantified using I^2^ and τ^2^ statistics.

#### 2.8.3. Exploratory Semi-Quantitative Measures and PET Thresholds

Where sufficient studies reported semi-quantitative parameters, we calculated pooled estimates using random-effects models. Given the exploratory nature and anticipated methodological heterogeneity, these analyses were interpreted cautiously and presented as hypothesis-generating. Where meta-analysis was unable to be performed due to few available studies, findings were discussed qualitatively.

#### 2.8.4. Handling of Between-Study Heterogeneity

Substantial heterogeneity was anticipated across studies due to variations in positivity thresholds for ^18^F-FDG PET (visual assessment, semi-quantitative SUV-based cutoffs, qualitative scoring systems), definitions of target condition, and reference standard components (clinical assessment, microbiological confirmation, inflammatory markers, imaging follow-up).

### 2.9. Assessing Risk of Bias and Applicability

Risk of bias and concerns regarding applicability were assessed independently by two reviewers (ML and MR) using the Quality Assessment of Diagnostic Accuracy Studies-2 (QUADAS-2) tool (University of Bristol, Bristol, UK) [42]. Disagreements were resolved through consensus.

Assessment of publication bias using contour-enhanced funnel plots and Egger’s regression test was planned if at least 10 studies were available per analysis, recognising the limited power and interpretability of such methods in diagnostic accuracy meta-analyses.

## 3. Results

### 3.1. Study Selection

**Figure 1:**
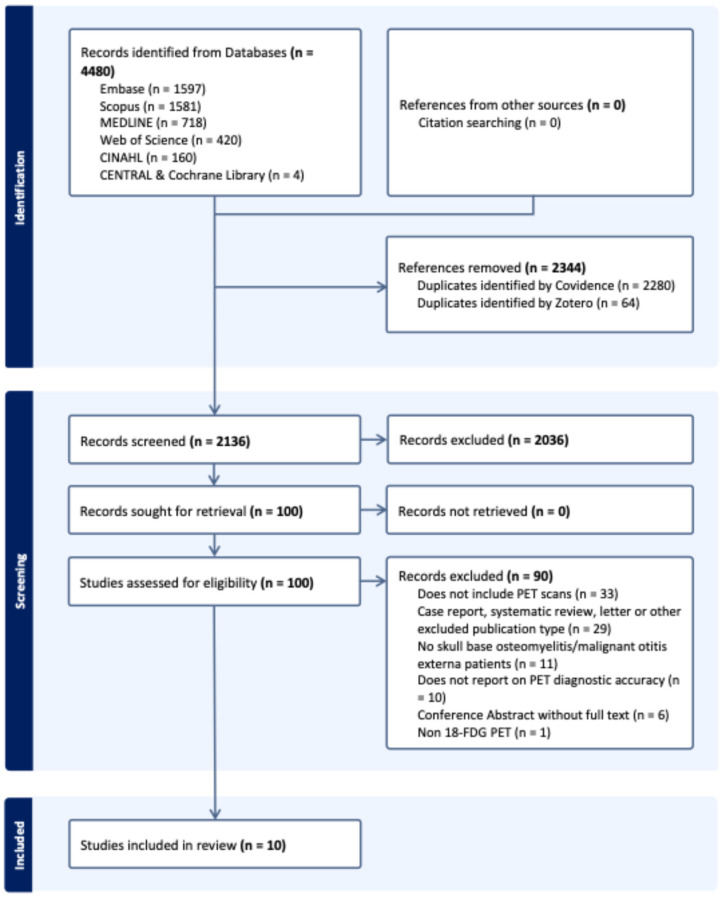
PRISMA flow diagram showing study selection process. Figure made using Covidence [34].

The search identified 4480 records in total from electronic databases, with no additional records from citation searching. After deduplication, 2136 unique records underwent title and abstract screening, of which 2036 were excluded. Full texts were sought for 100 records, of which 90 were excluded. In total, 10 studies were included in the review. The study selection process is depicted in the PRISMA flow diagram (Figure 1), and the full electronic search strategies are provided in Supplementary Data Section S1.

### 3.2. Study Characteristics

Ten studies (154 lesions for treatment response monitoring and 23 lesions for detection rate assessment) were included in the review and are detailed in Table 1. Nine were retrospective cohort studies, and one was prospective, with publication years ranging from 2019 to 2025, and they collectively represented Level II to IV evidence according to the Oxford Centre for Evidence-Based Medicine [43].

Eight studies provided extractable 2 × 2 diagnostic accuracy data for the primary outcome (treatment response monitoring) [33,44,45,46,47,48,49,50], whilst two studies [51,52] contributed only to the secondary outcome (detection rate at initial presentation). Participants’ ages ranged from 60.9 to 77 years (mean or median), with male predominance across all studies (67% to 96% male participants).

Reference standards were composite and varied across studies, incorporating combinations of clinical assessment (symptom resolution, otoscopic findings, cranial nerve function), biological markers (CRP, leucocyte count), microbiological confirmation, histopathology, and other imaging modalities.

### 3.3. Primary Outcome: Treatment Response Monitoring

**Figure 2:**
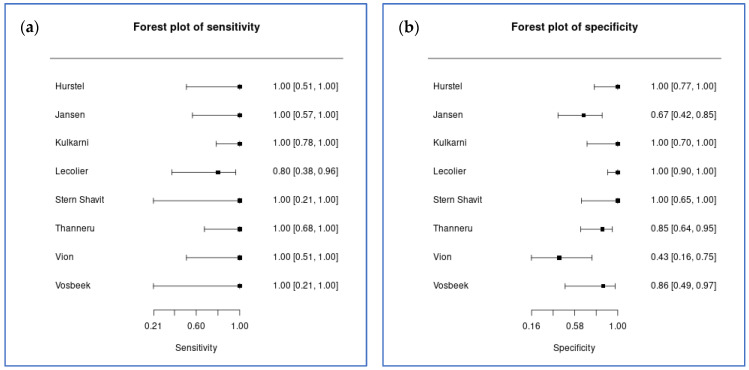
Coupled forest plots for treatment response monitoring (8 studies). (**a**) Sensitivity estimates with 95% confidence intervals; (**b**) specificity estimates with 95% confidence intervals.

Eight studies comprising 154 lesions contributed to the bivariate meta-analysis of ^18^F-FDG PET diagnostic accuracy for treatment response monitoring in SBO and NOE. Bivariate random-effects meta-analysis yielded a pooled sensitivity of 95.2% (95% credible interval [CrI] 85.6% to 99.0%) and a pooled specificity of 89.1% (95% CrI 70.7% to 96.7%) (Figure 2). The diagnostic odds ratio was 172.0, with a large confidence interval (95% CrI 28.6 to 1148.6). The positive likelihood ratio was 8.7 (95% CrI 3.2 to 28.4), and the negative likelihood ratio was 0.05 (95% CrI 0.01 to 0.17).

**Figure 3:**
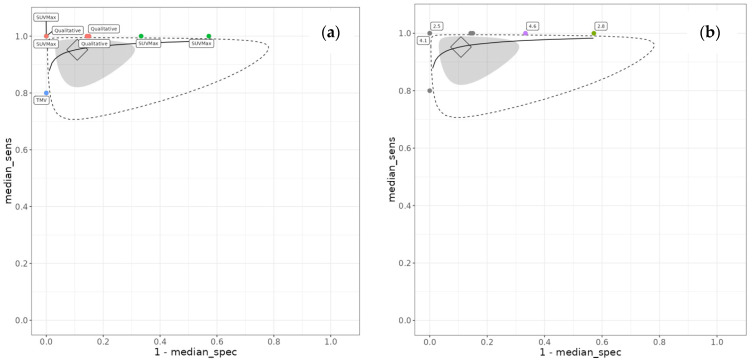
Summary receiver operating characteristic (SROC) curve for treatment response monitoring. (**a**) SROC with the FDG PET threshold method labelled. (**b**) SROC with SUVMax threshold labelled for the studies that used it as a diagnostic threshold. Diamond is the summary point (the pooled effect), the grey region is the 95% credible interval, and the dotted line represents the prediction interval. The solid black line is the extrapolated curve between the studies. The vertical axis, median_sens, represents the true positive rate, and the horizontal axis represents the false positive rate.

Between-study heterogeneity was substantial, particularly for specificity. The between-study standard deviation for logit-transformed specificity was 1.47 (95% CrI 0.61 to 2.56), whilst that for logit-transformed sensitivity was 0.59 (95% CrI 0.04 to 1.92). The between-study correlation was −0.06 (95% CrI −0.83 to 0.80), indicating minimal correlation between sensitivity and specificity across studies. The summary receiver operating characteristic curve is presented in Figure 3. The full output parameters are detailed in Supplementary Data Section S2.

### 3.4. Secondary Outcome: Diagnostic Detection Proportion at Initial Presentation

**Figure 4:**
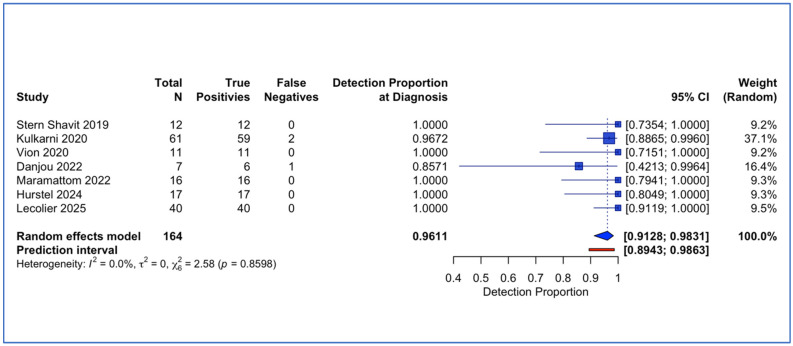
Forest plot of detection proportion at diagnosis [33,44,46,47,49,51,52].

Seven studies comprising 164 lesions (161 with positive PET findings) contributed to the meta-analysis of diagnostic detection proportion at initial presentation. Random-effects meta-analysis yielded a pooled detection rate of 96.1% (95% confidence interval [CI] 91.3% to 98.3%) (Figure 4). Individual study detection proportions ranged from 85.7% [52] to 100.0%. Between-study heterogeneity was minimal, with I^2^ = 0.0% (95% CI 0.0% to 70.8%), τ^2^ = 0 (95% CI 0.00 to 1.47), and Cochran’s Q = 2.58 (df = 6, *p* = 0.86).

### 3.5. Secondary Outcome: Semi-Quantitative Metabolic Parameters

Quantitative threshold approaches varied substantially, with most employing SUVmax-based metrics combined with visual assessment. Hurstel et al. (2024) used lesion-to-background ratios with 15 mm volumes of interest applied to affected and contralateral sides, achieving 100% accuracy for predicting recovery at a post-treatment ratio of 4.1; their initial diagnostic scan ratio of 4.73 provided 100% specificity for identifying poor responders [44]. Jansen et al. (2025) employed SUVpeak and SUVmax thresholds of 3.06 and 4.06 respectively (100% sensitivity, 67% specificity), applying an SUV threshold of 2.5 within manually defined portions of scans to define volumes of interest for volumetric calculations [45]. Lécolier et al. (2025) combined visual classification using hepatic SUVmax as reference (complete response ≤ hepatic activity, partial response > hepatic activity) with a novel 5-point intensity score across 12 anatomical regions generating a weighted “extent score”, though acknowledged limitations including unequal regional weighting, time-consuming application, and uncertain inter-observer reproducibility [46].

Volumetric and percentage change parameters were explored as complementary metrics. Lécolier et al. (2025) reported that total lesion glycolysis (TLG, metabolic volume × mean SUV) and total metabolic volume (TMV, voxels > 4.0 g/mL) achieved optimal discrimination: TLG ≥ 50.1 g and TMV ≥ 8.3 mL both yielded 80% sensitivity with 100% specificity for predicting relapse [46]. Percentage reductions (ΔTLG ≤ −37.4%, ΔTMV ≤ −45.5%, ΔExtent ≤ −4.3%) demonstrated 80% sensitivity with 82.9–97.1% specificity. Importantly, baseline disease burden (TLG, TMV, extent) significantly predicted relapse (*p* ≤ 0.003), whilst initial SUVmax did not (*p* = 0.968). Conversely, Jansen et al. (2025) found no volumetric associations with recurrence, suggesting focal pathogen eradication detected by SUVpeak was more critical than overall metabolic volume reduction [45].

Causative organisms demonstrated associations with SUV intensity and outcomes. Kulkarni et al. (2020) reported significantly higher SUVmax for bacterial (7.93) and mixed (7.78) versus fungal infections (4.38, *p* < 0.001) [31]. Despite lower metabolic activity, Danjou et al. (2022) identified fungal infection (*Aspergillus flavus*) as an independent relapse risk factor (adjusted hazard ratio 4.1, 95% CI 1.1–15, *p* = 0.03) [52].

### 3.6. Risk of Bias

**Figure 5:**
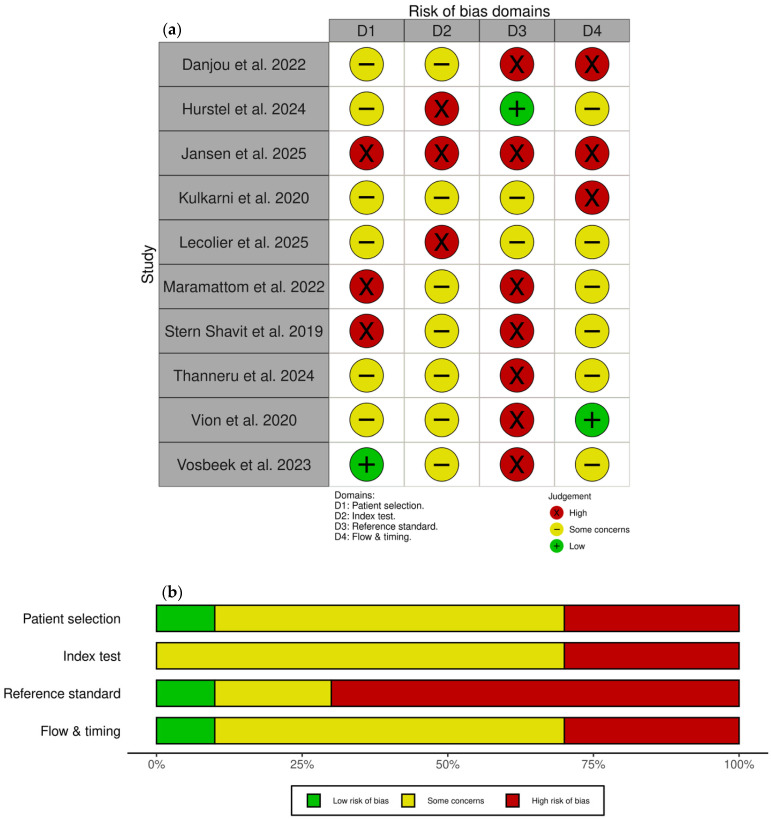
Risk of bias and applicability assessment using QUADAS-2 tool. (**a**) Traffic light plot showing risk of bias judgements for each included study across four domains [33,44,45,46,47,48,49,50,51,52]; (**b**) weighted bar chart showing proportions of studies with low, some concerns, and high risk of bias for each domain. Figures generated using the robvis tool [53].

Risk of bias and applicability assessments using the QUADAS-2 tool are presented in Figure 5. Overall, the risk of bias was medium to high across all domains, with only three studies achieving low risk for any individual domain. Common concerns included retrospective selection without disease-free controls, lack of prospectively defined PET positivity thresholds, unclear blinding, and variable composite reference standards without prespecified definitions. Potential incorporation bias, where PET findings may have influenced clinical management decisions forming part of the reference standard, was identified in two studies.

Signalling questions were tailored to the review context and are provided in Supplementary Data Section S3.

Assessment of publication bias was not feasible as fewer than 10 studies contributed to each meta-analysis (treatment response monitoring, *n* = 8; detection rate, *n* = 7), falling below the recommended threshold for reliable evaluation in diagnostic test accuracy meta-analyses.

## 4. Discussion

This systematic review and meta-analysis evaluated the diagnostic test accuracy of ^18^F-FDG PET for treatment response monitoring in skull base osteomyelitis and necrotising otitis externa. It also explored detection accuracy at the point of diagnosis. Eight studies comprising 154 lesions contributed to the primary outcome analysis, yielding a pooled sensitivity of 95.2% (95% credible interval 85.6% to 99.0%) and a pooled specificity of 89.1% (95% credible interval 70.7% to 96.7%). The positive likelihood ratio was 8.7 (95% credible interval 3.2 to 28.4) and the negative likelihood ratio was 0.05 (95% credible interval 0.01 to 0.17), with a diagnostic odds ratio of 172.0 (95% credible interval 28.6 to 1148.6).

From a clinical perspective, these findings indicate that ^18^F-FDG PET demonstrates excellent capacity to rule out persistent or recurrent active infection during treatment monitoring. The very low negative likelihood ratio of 0.05 indicates that a negative PET result substantially decreases the probability of ongoing active infection, supporting safe treatment cessation decisions. Conversely, whilst the positive likelihood ratio of 8.7 represents moderate to good diagnostic utility, the specificity of 89.1% suggests that positive PET findings should be interpreted in conjunction with clinical and laboratory parameters, as false-positive results may occur in approximately 11% of cases. This likely reflects the challenge of distinguishing residual metabolic activity from post-inflammatory changes during the healing process, particularly given the anatomical complexity of the skull base and the variable time course of metabolic normalisation following infection resolution.

Our findings are comparable to published meta-analyses evaluating ^18^F-FDG PET diagnostic accuracy for osteomyelitis at other anatomical sites. Prior systematic reviews of general osteomyelitis and diabetic foot osteomyelitis reported pooled sensitivity ranging from 74% to 96% and specificity from 91% to 92.8% [24,28,29,30]. Importantly, none of these prior systematic reviews included skull base osteomyelitis or necrotising otitis externa, making the current analysis the first meta-analysis specifically addressing diagnostic test accuracy of ^18^F-FDG PET in this anatomical location. Our pooled sensitivity of 95.2% positions it at the upper end of reported values, suggesting that ^18^F-FDG PET performs particularly well for treatment response assessment in skull base infection.

The secondary outcome analysis evaluated diagnostic detection rate at initial presentation across seven studies comprising 164 lesions with confirmed skull base osteomyelitis, yielding a pooled rate of 96.1% (95% confidence interval 91.3% to 98.3%) with minimal heterogeneity. However, this finding must be interpreted with caution as six of the seven studies enrolled only patients with already-confirmed disease, lacking disease-free control groups necessary for calculating true diagnostic sensitivity and specificity. This analysis should therefore be considered exploratory and hypothesis-generating rather than definitive evidence of diagnostic accuracy. Notably, Kulkarni et al. (2020) was the sole study evaluating ^18^F-FDG PET in patients with suspected skull base osteomyelitis including appropriate controls, reporting a sensitivity of 96.7%, a specificity of 93.8%, a positive predictive value of 98.3%, and a negative predictive value of 88.2% [31]. These encouraging results from the only methodologically robust diagnostic accuracy study at the diagnosis timepoint, combined with the high positivity rate observed in confirmed cases, suggest that ^18^F-FDG PET may have substantial value for diagnostic evaluation at initial presentation, warranting further investigation in appropriately designed prospective studies.

^18^F-FDG PET offers several advantages over alternative imaging modalities for skull base osteomyelitis assessment. Conventional CT demonstrates persistent bone changes that emerge late and that fail to normalise following infection resolution, whilst MRI findings lag behind clinical improvement and may remain abnormal for extended periods [11,18,19,20]. Technetium-99m bone scintigraphy remains positive during prolonged bone remodelling phases, and gallium-67 imaging suffers from limited spatial resolution and restricted availability [22,23,24,25]. These imaging modalities demonstrate persistent abnormalities extending well beyond active disease resolution, confounding determination of the treatment endpoint. In contrast, ^18^F-FDG PET directly images glucose metabolism in activated inflammatory cells, potentially normalising at a rate concordant with disease resolution. Furthermore, ^18^F-FDG PET/CT infrastructure is widely available in most healthcare systems due to its established role in oncological imaging, providing accessibility without requiring specialised radiopharmaceutical production or dedicated infection imaging programmes. Compared to ^18^F-FDG-labelled autologous leukocyte imaging, standard ^18^F-FDG PET is considerably simpler and faster to perform, not requiring blood withdrawal, ex vivo cell labelling, or extended preparation times [54].

Recent studies demonstrate that ^18^F-FDG PET is successfully guiding clinical management in skull base osteomyelitis and necrotising otitis externa, though standardised thresholds remain undefined. A recent prospective trial achieved significant antibiotic de-escalation using PET/CT at 3 months post-treatment, reducing median oral antibiotic duration from 40 to 8 weeks without increased relapse [55]. In a retrospective study of 33 patients, the findings suggested a potential reduction in the treatment duration of 48% of patients based on percentage SUVmax reductions [21]. A consistent challenge is managing partial responders. For approximately 50% of patients in these studies, neither complete resolution nor progression was evident, underscoring the need for validated, imaging parameter threshold values and standardised response criteria [21,55,56].

### 4.1. Limitations

This systematic review has several important limitations. Risk of bias assessment using QUADAS-2 identified medium to high risk across most domains, with only two studies achieving low risk for any individual domain. Common methodological concerns included retrospective patient selection without disease-free controls, lack of prospectively defined PET positivity thresholds, unclear blinding of PET readers to reference standard results, and potential incorporation bias, where PET findings may have influenced clinical management decisions. Substantial heterogeneity was observed for specificity estimates, with between-study standard deviation on the logit scale of 1.47 (95% credible interval 0.61 to 2.56), likely reflecting variability in PET positivity criteria (five studies employed semiquantitative parameters, three used qualitative visual assessment) and composite reference standards without clearly prespecified criteria. Accordingly, the pooled estimates, particularly specificity and the diagnostic odds ratio, should be interpreted as average effects with substantial uncertainty and may not be directly generalisable to all clinical contexts.

Our primary analysis was conducted per lesion because patient-level clustering could not be consistently reconstructed from published data; while most cohorts were unilateral, any unaccounted within-patient correlation (e.g., bilateral or contralateral disease) may underestimate statistical uncertainty in the pooled estimates.

The absence of a universally accepted gold standard for treatment response assessment in skull base osteomyelitis represents an inherent challenge in assessing diagnostic test accuracy. Publication bias could not be formally assessed as fewer than 10 studies contributed to each meta-analysis. The relatively small number of included studies (*n* = 8) and modest sample size (*n* = 154 lesions) limit the precision of pooled estimates, reflected in wide credible intervals for specificity and diagnostic odds ratio.

Notably, disease-specific consensus guidance and validated, standardised PET response criteria to define treatment endpoints in SBO/NOE remain limited. This likely contributes to variability in thresholds and composite reference standards across studies and institutions.

Finally, included cohorts predominantly represented NOE-related (otogenic) SBO, with fewer studies enrolling broader or central SBO. Differences in diagnostic pathways and composite reference standards between these subtypes may influence estimates and generalisability; the limited number of studies per subtype precluded robust subtype-specific comparisons of diagnostic performance.

### 4.2. Future Research

Prospective diagnostic test accuracy studies of ^18^F-FDG PET in skull base osteomyelitis are needed for more definitive conclusions to be made. Future studies should employ prospective designs with prespecified, validated PET positivity thresholds and well-defined reference standards. Blinding of PET readers to reference standard results is essential to minimise interpretation bias. Critically, future research should evaluate ^18^F-FDG PET performance in patients with suspected skull base osteomyelitis at initial clinical presentation, including disease-free control groups to enable calculation of true diagnostic sensitivity and specificity. The 96.1% detection rate identified in this review, alongside encouraging results from one study evaluating suspected cases [33], suggests such studies would be both safe and feasible, potentially enabling earlier therapeutic intervention and more accurate timing of cessation of treatment.

Investigation of semi-quantitative PET parameters (SUVmax, SUVpeak, total metabolic volume) as prognostic markers would be of considerable interest. Future studies should prospectively evaluate whether specific metabolic thresholds at diagnosis or during treatment can predict clinical outcomes, including treatment failure, disease recurrence, or relapse. Systematic analysis would help establish optimal cutoff values for clinical decision-making and identify relationships between PET parameters and causative pathogens, given preliminary evidence suggesting fungal infections demonstrate lower ^18^F-FDG uptake compared to bacterial infections [33,52,57]. Such prognostic markers could enable risk stratification and guide individualised treatment duration. Furthermore, the use of the same imaging modality at both diagnosis and treatment response timepoints would allow examination of the prognostic and diagnostic value of changes in PET parameters rather than the absolute values of these parameters alone.

## 5. Conclusions

This systematic review provides the first meta-analysis evidence that ^18^F-FDG PET demonstrates high sensitivity and good specificity for treatment response monitoring in skull base osteomyelitis and necrotising otitis externa. The excellent negative likelihood ratio supports the use of ^18^F-FDG PET to guide safe treatment cessation decisions, addressing an unmet need in clinical practice where premature antibiotic discontinuation risks devastating relapse. However, the evidence base is limited by small sample sizes, retrospective study designs, and substantial risk of bias. Prospective diagnostic test accuracy studies with prespecified PET positivity thresholds, appropriate blinding, and well-defined reference standards are needed to validate these findings and establish the role of ^18^F-FDG PET in both treatment monitoring and initial diagnosis of skull base osteomyelitis. Investigation of semi-quantitative metabolic parameters as prognostic markers represents a promising avenue for future research.

## Figures and Tables

**Table 1:** Study Characteristics of Included Studies.

Study	Setting	Design	Country	Study Period	OCEBM Level	Index Test	Reference Test	Target Condition	Age (Years)	Sex (% Male)	Diagnosis Timepoint			Follow Up Timepoint				
											N	TP	FN	N	TP	FP	TN	FN
Kulkarni 2020 [31]	Single-centre	Retrospective cohort	India	2012–2019	3	PET/CT	Histopathology (infective granulation/necrosis) or culture positivity or clinical improvement (symptoms/inflammatory markers) with antimicrobials OR imaging response	SBO	66.4 ± 9.4 (45–92)	0.73	61	59	2	23	14	0	9	0
Hurstel 2024 [44]	Single-centre	Retrospective cohort	France	2020–2023	3	PET/CT	Clinical/otoscopic/biological recovery (normalised CRP) at end of treatment + 3-month follow-up confirmation, ENT physicians blinded to imaging	NOE	74.0 ± 10.6 (median 72.0, IQR 62.5–78)	0.71	17	17	0	17	4	0	13	0
Jansen 2025 [45]	Single-centre	Retrospective cohort	The Netherlands	2011–2022	3	PET/CT	Recurrent disease defined as both recurrent symptoms (otalgia, otorrhea, granulation/inflammation, or cranial nerve palsy) and unchanged/progressed imaging on CT, MRI, or PET/CT, minimum 6-month follow-up	NOE	77 (54–91) mean (range)	0.8	—	—	—	20	5	5	10	0
Lécolier 2025 [46]	Single-centre	Retrospective cohort	France	2016–2024	3	PET/CT	Clinical examination at re-evaluation + minimum 3-month follow-up for absence of early recurrence. Complete clinical response = complete EAC healing on otoscopy + resolution of symptoms (excluding facial paralysis)	NOE	76 ± 9 (52–91)	0.816	40	40	0	40	4	0	35	1
Stern Shavit 2019 [47]	Single-centre	Retrospective cohort	Israel	2013–2017	4	PET/CT	Clinical criteria (external otitis with severe otalgia, EAC oedema/exudate/granulations + failure to respond to systemic/local antibiotics ≥ 1 week + histology compatible with inflammation)	NEO	74 ± 11.5	0.83	12	12	0	8	1	0	7	0
Thanneru 2025 [48]	Single-centre	Prospective cohort	India	2021–2023	2	PET/CT	Clinical resolution = asymptomatic status maintained without new signs of disease for minimum 3 weeks after stopping treatment	NOE	60.9 ± 14.3	0.964	—	—	—	28	8	3	17	0
Vion 2020 [49]	Single-centre	Retrospective cohort	France	Not specified	3	PET/CT	Combination of clinical (persistent otalgia/otorrhea, cranial nerve palsy) + biological (leucocyte count/CRP) criteria. Complete response confirmed retrospectively by absence of relapse during ≥ 12-month follow-up	SBO	72 ± 9	0.9	11	11	0	11	4	4	3	0
Vosbeek 2023 [50]	Single-centre	Retrospective cohort	The Netherlands	2013–2022	3	PET/CT	Clinical relapse during ≥ 3 months follow-up after therapy cessation. Relapse = progression of symptoms after symptom-free period after therapy cessation	NOE	75 (43–91)	0.83	—	—	—	7	1	1	5	0
Maramattom 2022 [51]	Single-centre	Retrospective cohort	India	2015–2020	3	PET/CT	Clinical (headache, cranial neuropathy, scalp tenderness) + imaging (MRI, CT, bone scintigraphy) + endoscopy-guided biopsy with microbiology	CSBO	>50 (52–73) range	0.76	16	16	0	—	—	—	—	—
Danjou 2022 [52]	Single-centre	Retrospective cohort	France	2006–2018	3	PET	Clinical + imaging (bone scintigraphy/MRI/CT/PET)	NOE	75 (69–81) median (IQR)	0.67	7	6	1	—	—	—	—	—

Abbreviations: —, no data for; CSBO, central skull base osteomyelitis; CRP, C-reactive protein; CT, computed tomography; EAC, external auditory canal; ENT, ear, nose, and throat; FN, false negative; FP, false positive; IQR, interquartile range; MRI, magnetic resonance imaging; N, number; NEO, necrotising external otitis; NOE, necrotising otitis externa; OCEBM, Oxford Centre for Evidence-Based Medicine; PET, positron emission tomography; PET/CT, positron emission tomography/computed tomography; SBO, skull base osteomyelitis; TN, true negative; TP, true positive.

## Data Availability

The raw data supporting the conclusions of this article are available in the article text (Table 1) and in the available Supplementary Data.

## Supplementary Materials

The following supporting information can be downloaded at: https://www.mdpi.com/article/10.3390/tomography12030032/s1, Section S1: Systematic Search Strategy; Section S2: MetaBayesDTA Full Statistical Output; Section S3: Risk of Bias QUADAS-2 Signalling Questions; Section S4: PRISMA DTA for Abstracts Checklist.

## Abbreviations

The following abbreviations are used in this manuscript:

^18^F-FDG PET	Fluorine-18-fluorodeoxyglucose Positron Emission Tomography
ASOHNS	Australasian Society of Otolaryngology Head and Neck Surgery
CENTRAL	Cochrane Central Register of Controlled Trials
CI	Confidence Interval
CINAHL	Cumulative Index to Nursing and Allied Health Literature
CrI	Credible Interval
CRP	C-Reactive Protein
CSBO	Central Skull Base Osteomyelitis
CT	Computed Tomography
EAC	External Auditory Canal
ENT	Ear, Nose, and Throat
FN	False Negative
FP	False Positive
Ga-67	Gallium-67
GLUT	Glucose Transporter
IQR	Interquartile Range
MOE	Malignant Otitis Externa
MRI	Magnetic Resonance Imaging
NEO	Necrotising External Otitis
NOE	Necrotising Otitis Externa
OCEBM	Oxford Centre for Evidence-Based Medicine
PET/CT	Positron Emission Tomography/Computed Tomography
PET/MRI	Positron Emission Tomography/Magnetic Resonance Imaging
PICO	Population, Index test, Comparator, and Outcomes
PRISMA-DTA	Preferred Reporting Items for Systematic Review and Meta-Analyses of Diagnostic Test Accuracy
PROSPERO	International Prospective Register of Systematic Reviews
QUADAS-2	Quality Assessment of Diagnostic Accuracy Studies-2
RACS	Royal Australasian College of Surgeons
SBO	Skull Base Osteomyelitis
SPECT	Single Photon Emission Computed Tomography
SROC	Summary Receiver Operating Characteristic
SUV	Standardised Uptake Value
SUVmax	Maximum Standardised Uptake Value
SUVpeak	Peak Standardised Uptake Value
Tc-99m	Technetium-99m
TLG	Total Lesion Glycolysis
TMV	Total Metabolic Volume
TN	True Negative
TP	True Positive
