# Supplementary material for: Diagnostic Test Accuracy and Semi-Quantitative Metrics of 18F-FDG PET in Assessing Treatment Response in Skull Base Osteomyelitis and Necrotising Otitis Externa: A Systematic Review and Meta-Analysis
Source: Tomography. 2026 Mar 2;12(3):32. doi: 10.3390/tomography12030032 (PMC13030614; doi:10.3390/tomography12030032)
Supplement: Supplementary file 1 [file tomography-12-00032-s001.zip › Section 4-PRISMA DTA for Abstracts Checklist.pdf]

PRISMA-DTA for Abstracts Checklist

Section/topic	#	PRISMA-DTA for Abstracts Checklist item	Reported on line #
**TITLE and PURPOSE**			
Title	1	Identify the report as a systematic review (+/- meta-analysis) of diagnostic test accuracy (DTA) studies.	2-5
Objectives	2	Indicate the research question, including components such as participants, index test, and target conditions.	32-35
**METHODS**			
Eligibility criteria	3	Include study characteristics used as criteria for eligibility.	37-39
Information sources	4	List the key databases searched and the search dates.	36-37
Risk of bias & applicability	5	Indicate the methods of assessing risk of bias and applicability.	39-40
Synthesis of results	A1	Indicate the methods for the data synthesis.	40-41
**RESULTS**			
Included studies	6	Indicate the number and type of included studies and the participants and relevant characteristics of the studies (including the reference standard).	42,46
Synthesis of results	7	Include the results for the analysis of diagnostic accuracy, preferably indicating the number of studies and participants. Describe test accuracy including variability; if meta-analysis was done, include summary results and confidence intervals.	42-48
**DISCUSSION**			
Strengths and limitations	9	Provide a brief summary of the strengths and limitations of the evidence	48-52
Interpretation	10	Provide a general interpretation of the results and the important implications.	48-52
**OTHER**			
Funding	11	Indicate the primary source of funding for the review.	490
Registration	12	Provide the registration number and the registry name	108-109

Adapted From: McInnes MDF, Moher D, Thombs BD, McGrath TA, Bossuyt PM, The PRISMA-DTA Group (2018). Preferred Reporting Items for a Systematic Review and Meta-analysis of Diagnostic Test Accuracy Studies: The PRISMA-DTA Statement. JAMA. 2018 Jan 23;319(4):388-396. doi: 10.1001/jama.2017.19163.
