# Supplementary material for: Diagnostic Test Accuracy and Semi-Quantitative Metrics of 18F-FDG PET in Assessing Treatment Response in Skull Base Osteomyelitis and Necrotising Otitis Externa: A Systematic Review and Meta-Analysis
Source: Tomography. 2026 Mar 2;12(3):32. doi: 10.3390/tomography12030032 (PMC13030614; doi:10.3390/tomography12030032)
Supplement: Supplementary file 1 [file tomography-12-00032-s001.zip › Supplementary Data.pdf]

# Supplementary Data for

# Diagnostic Test Accuracy and Semi-Quantitative Metrics of $^{18}$F-FDG PET in Assessing Treatment Response in Skull Base Osteomyelitis and Necrotising Otitis Externa: A Systematic Review and Meta-Analysis

**Mark Laidlaw [1,*], Maya Reid [2], Sukanya Rajiv [1,*] and Jean-Marc Gerard [1,3,4]**

[1] Department of Otolaryngology, Royal Victorian Eye and Ear Hospital, Melbourne, VIC 3002, Australia
[2] Department of Otolaryngology, Head and Neck Surgery, Canberra Health Service, Canberra, ACT 2605, Australia
[3] Victorian Cochlear Implant Program, Royal Victorian Eye and Ear Hospital, Melbourne, VIC 3002, Australia
[4] Department of Otolaryngology, University of Melbourne, Melbourne, VIC 3010, Australia
* Correspondence: mark.laidlaw@eyeandear.org.au (M.L.); sukanya.rajiv@eyeandear.org.au (S.R.)

## Table of Contents

***Supplementary Data Section 1: Systematic Search Strategy*** ........................................................ **2**

***Supplementary Data Section 2: MetaBayesDTA Full Statistical Output*** ............................................ **8**

***Supplementary Data Section 3: Risk of Bias QUADAS-2 Signalling Questions*** ................................ **10**

# Supplementary Data Section S1: Systematic Search Strategy

## Ovid MEDLINE

Ovid MEDLINE(R) ALL <1946 to November 26, 2025>

| # | Search Query | Results |
|---|---|---|
| 1 | ((petr$ or skull or crani$ or invas$ or necroti$ or malig$) adj2 osteomy$).mp. [mp=title, book title, abstract, original title, name of substance word, subject heading word, floating sub-heading word, keyword heading word, organism supplementary concept word, protocol supplementary concept word, rare disease supplementary concept word, unique identifier, synonyms, population supplementary concept word, anatomy supplementary concept word] | 736 |
| 2 | ((necroti$ or malig$ or invas$) adj1 otit$ adj1 extern$).mp. | 522 |
| 3 | (Otit$ adj1 extern$).mp. [mp=title, book title, abstract, original title, name of substance word, subject heading word, floating sub-heading word, keyword heading word, organism supplementary concept word, protocol supplementary concept word, rare disease supplementary concept word, unique identifier, synonyms, population supplementary concept word, anatomy supplementary concept word] | 3948 |
| 4 | (Petrositis or Gradenigo).mp. [mp=title, book title, abstract, original title, name of substance word, subject heading word, floating sub-heading word, keyword heading word, organism supplementary concept word, protocol supplementary concept word, rare disease supplementary concept word, unique identifier, synonyms, population supplementary concept word, anatomy supplementary concept word] | 267 |
| 5 | Petrositis/ or (Malignant otitis externa or Otitis Externa).mp. or Otitis Externa/ | 3839 |
| 6 | 1 or 2 or 3 or 4 or 5 | 4751 |
| 7 | (Diagnostic Imaging or Tomography, Emission-Computed or Positron-Emission Tomography or Positron Emission Tomography Computed Tomography or Tomography or Tomography, Emission-Computed or Positron-Emission Tomography or Positron Emission Tomography Computed Tomography).mp. | 2116939 |
| 8 | (Positron?emission tomography or PET or PET?CT$ or CT?PET$ or Fluorodeoxyglucose F18 or Fluorodeoxyglucose or fludeoxyglucose or FDG or FDG?PET$ or Flu?rodeoxyglucose or 18?F?FDG?PET* or 2?F?18?F?FDG?PET*or 2-Flu?rine-18 or 18?F? or 18?F?Flu?rodeoxyglucos or 2?Flu?rine?18?Flu?rodeoxyglucose or Flu?rodeoxyglucose$).mp. [mp=title, book title, abstract, original title, name of substance word, subject heading word, floating sub-heading word, keyword heading word, organism supplementary concept word, protocol supplementary concept word, rare disease supplementary concept word, unique identifier, synonyms, population supplementary concept word, anatomy supplementary concept word] | 168029 |
| 9 | 7 or 8 | 2166089 |
| 10 | 6 and 9 | 718 |

## CINAHL

Search Mode: Proximity

Interface: EBSCOhost Research Databases

Database(s): CINAHL Complete

| # | Search Query | Results |
|---|---|---|
| S1 | Query (user-entered): ((petr* OR skull OR crani* OR invas* OR necroti* OR malig*) N2 osteomy*)<br>Query (expanded/display term): ((petr* OR skull OR crani* OR invas* OR necroti* OR malig*) N2 osteomy*) | 207 |
| S2 | Query (user-entered): ((necroti* OR malig* OR invas*) N1 otit* N1 extern*)<br>Query (expanded/display term): ((necroti* OR malig* OR invas*) N1 otit* N1 extern*) | 216 |
| S3 | Query (user-entered): (Otit* N1 extern*)<br>Query (expanded/display term): (Otit* N1 extern*) | 688 |
| S4 | Query (user-entered): (Petrositis OR Gradenigo)<br>Query (expanded/display term): (Petrositis OR Gradenigo) | 58 |
| S5 | Query (user-entered): (MH Petrositis)<br>Query (expanded/display term): (MH Petrositis) | 0 |
| S6 | Query (user-entered): ("Malignant otitis externa" OR "Otitis Externa") OR (MH "Otitis Externa")<br>Query (expanded/display term): ("Malignant otitis externa" OR "Otitis Externa") OR (MH "Otitis Externa") | 632 |
| S7 | Query (user-entered): ("Diagnostic Imaging" OR "Tomography, Emission-Computed" OR "Positron-Emission Tomography" OR "Positron Emission Tomography Computed Tomography" OR Tomography OR "Tomography, Emission-Computed" OR "Positron-Emission Tomography" OR "Positron Emission Tomography Computed Tomography")<br>Query (expanded/display term): ("Diagnostic Imaging" OR "Tomography, Emission-Computed" OR "Positron-Emission Tomography" OR "Positron Emission Tomography Computed Tomography" OR Tomography OR "Tomography, Emission-Computed" OR "Positron-Emission Tomography" OR "Positron Emission Tomography Computed Tomography") | 234534 |
| S8 | Search Name: "Fluorodeoxyglucose F18" OR Fluorodeoxyglucose OR fludeoxyglucose<br>Query (user-entered): "Fluorodeoxyglucose F18"<br>Query (expanded/display term): "Fluorodeoxyglucose F18" | 13 |
| S9 | Query (user-entered): ("Positron#emission tomography" OR PET OR PET#CT* OR CT#PET* OR FDG OR FDG#PET* OR Flu#rodeoxyglucose OR 2-Flu#rine-18 OR 18#F# OR 18#F#Flu#rodeoxyglucos OR 2#Flu#rine#18#Flu#rodeoxyglucose OR Flu#rodeoxyglucose*)<br>Query (expanded/display term): ("Positron#emission tomography" OR PET OR PET#CT* OR CT#PET* OR FDG OR FDG#PET* OR Flu#rodeoxyglucose OR 2-Flu#rine-18 OR 18#F# OR 18#F#Flu#rodeoxyglucos OR 2#Flu#rine#18#Flu#rodeoxyglucose OR Flu#rodeoxyglucose*) | 35575 |
| S10 | Query (user-entered): ((S1) OR (S2) OR (S3) OR (S4) OR (S5) OR (S6))<br>Query (expanded/display term): (((((petr* OR skull OR crani* OR invas* OR necroti* OR malig*) N2 osteomy*))) OR ((((necroti* OR malig* OR invas*) N1 otit* N1 extern*))) OR (((Otit* N1 extern*))) OR (((Petrositis OR Gradenigo))) OR (((MH Petrositis))) OR ((("Malignant otitis externa" OR "Otitis Externa") OR (MH "Otitis Externa")))) | 903 |
| S11 | Query (user-entered): (S7) OR (S8) OR (S9)<br>Query (expanded/display term): ((("Diagnostic Imaging" OR "Tomography, Emission-Computed" OR "Positron-Emission Tomography" OR "Positron Emission Tomography Computed Tomography" OR Tomography OR "Tomography, Emission-Computed" OR | 252854 |

| | | |
|---|---|---|
| | "Positron-Emission Tomography" OR "Positron Emission Tomography Computed Tomography"))) OR (("Fluorodeoxyglucose F18")) OR ((("Positron#emission tomography" OR PET OR PET#CT* OR CT#PET* OR FDG OR FDG#PET* OR Flu#rodeoxyglucose OR 2-Flu#rine-18 OR 18#F# OR 18#F#Flu#rodeoxyglucos OR 2#Flu#rine#18#Flu#rodeoxyglucose OR Flu#rodeoxyglucose*))) | |
| S12 | Query (user-entered): (S10) AND (S11)<br>Query (expanded/display term): (((((((petr* OR skull OR crani* OR invas* OR necroti* OR malig*) N2 osteomy*))) OR ((((necroti* OR malig* OR invas*) N1 otit* N1 extern*))) OR (((Otit* N1 extern*))) OR (((Petrositis OR Gradenigo))) OR (((MH Petrositis))) OR ((("Malignant otitis externa" OR "Otitis Externa") OR (MH "Otitis Externa")))))) AND ((((("Diagnostic Imaging" OR "Tomography, Emission-Computed" OR "Positron-Emission Tomography" OR "Positron Emission Tomography Computed Tomography" OR Tomography OR "Tomography, Emission-Computed" OR "Positron-Emission Tomography" OR "Positron Emission Tomography Computed Tomography"))) OR (("Fluorodeoxyglucose F18")) OR ((("Positron#emission tomography" OR PET OR PET#CT* OR CT#PET* OR FDG OR FDG#PET* OR Flu#rodeoxyglucose OR 2-Flu#rine-18 OR 18#F# OR 18#F#Flu#rodeoxyglucos OR 2#Flu#rine#18#Flu#rodeoxyglucose OR Flu#rodeoxyglucose*))))) | 160 |

## Cochrane (CSDR and Central)

| # | Search Query | Results |
|---|---|---|
| 1 | ((petr*:ti,ab,kw OR skull:ti,ab,kw OR crani*:ti,ab,kw OR invas*:ti,ab,kw OR necroti*:ti,ab,kw OR malig*:ti,ab,kw) NEAR/2 osteomy*:ti,ab,kw) | 11 |
| 2 | ((necroti*:ti,ab,kw OR malig*:ti,ab,kw OR invas*:ti,ab,kw) NEAR/1 otit*:ti,ab,kw NEAR/1 extern*:ti,ab,kw) | 3 |
| 3 | (MOE:ti,ab,kw OR NOE:ti,ab,kw) | 95 |
| 4 | (Otit*:ti,ab,kw NEAR/1 extern*:ti,ab,kw) | 277 |
| 5 | (Petrositis:ti,ab,kw OR Gradenigo:ti,ab,kw) | 3 |
| 6 | [mh ^Petrositis] | 0 |
| 7 | ("Malignant otitis externa":ti,ab,kw OR "Otitis Externa":ti,ab,kw) OR [mh ^"Otitis Externa"] | 224 |
| 8 | #1 OR #2 OR #3 OR #4 OR #5 OR #6 OR #7 | 384 |
| 9 | ("Diagnostic Imaging":ti,ab,kw OR "Tomography, Emission-Computed":ti,ab,kw OR "Positron-Emission Tomography":ti,ab,kw OR "Positron Emission Tomography Computed Tomography":ti,ab,kw OR Tomography:ti,ab,kw OR "Tomography, Emission-Computed":ti,ab,kw OR "Positron-Emission Tomography":ti,ab,kw OR "Positron Emission Tomography Computed Tomography":ti,ab,kw) | 75228 |
| 10 | "Fluorodeoxyglucose F18":ti,ab,kw | 958 |
| 11 | ((Positron?emission NEXT "tomography"):ti,ab,kw OR PET:ti,ab,kw OR PET?CT*:ti,ab,kw OR CT?PET*:ti,ab,kw OR FDG:ti,ab,kw OR FDG?PET*:ti,ab,kw OR Flu?rodeoxyglucose:ti,ab,kw OR 18?F?:ti,ab,kw OR 18?F?Flu?rodeoxyglucos:ti,ab,kw OR 2?Flu?rine?18?Flu?rodeoxyglucose:ti,ab,kw OR Flu?rodeoxyglucose*:ti,ab,kw) | 10943 |
| 12 | #9 OR #10 OR #11 | 79927 |
| 13 | #8 AND #12 | 4 |

## Embase

| # | Search Query | Results |
|---|---|---|
| 1 | (petr* OR skull OR crani* OR invas* OR necroti* OR malig*) NEAR/2 osteomy* | 985 |
| 2 | (necroti* OR malig* OR invas*) NEAR/1 otit* NEAR/1 extern* | 1163 |
| 3 | otit* NEAR/1 extern* | 6230 |
| 4 | petrositis OR gradenigo | 1881 |
| 5 | 'petrositis'/de OR 'malignant otitis externa' OR 'otitis externa' OR 'otitis externa'/de | 6220 |
| 6 | #1 OR #2 OR #3 OR #4 OR #5 | 8803 |
| 7 | 'diagnostic imaging' OR tomography OR 'tomography, emission-computed' OR 'positron-emission tomography' OR 'positron emission tomography computed tomography' | 2084559 |
| 8 | 'positron$emission tomography' OR pet OR pet$ct* OR ct$pet* OR fluorodeoxyglucose f18' OR fluorodeoxyglucose OR fludeoxyglucose OR fdg OR fdg$pet* OR flu$rodeoxyglucose OR 18$f$fdg$pet* OR '2$f$18$f$fdg$pet*or 2-flu$rine-18' OR 18$f$ OR 18$f$flu$rodeoxyglucos OR 2$flu$rine$18$flu$rodeoxyglucose OR flu$rodeoxyglucose* | 353193 |
| 9 | #7 OR #8 | 2191543 |
| 10 | #6 AND #9 | 1597 |

## Scopus

| # | Search Query | Results |
|---|---|---|
| 1 | TITLE-ABS-KEY ( ( petr* or skull or crani* or invas* or necroti* or malig* ) W/2 osteomy* ) | 1209 |
| 2 | TITLE-ABS-KEY ( ( necroti* or malig* or invas* ) W/1 otit* W/1 extern* ) | 1151 |
| 3 | TITLE-ABS-KEY ( otit* W/1 extern* ) | 6628 |
| 4 | TITLE-ABS-KEY ( petrositis OR gradenigo ) | 549 |
| 5 | INDEXTERMS ( petrositis ) OR TITLE-ABS-KEY ( "malignant otitis externa" OR "otitis externa" ) ORINDEXTERMS ( "otitis externa" ) | 4743 |
| 6 | ( TITLE-ABS-KEY ( ( petr* or skull or crani* or invas* or necroti* or malig* ) W/2 osteomy* ) ) OR ( TITLE-ABS-KEY ( ( necroti* or malig* or invas* ) W/1 otit* W/1 extern* ) ) OR ( TITLE-ABS-KEY ( otit* W/1 extern* ) ) OR (TITLE-ABS-KEY ( petrositis OR gradenigo ) ) OR ( INDEXTERMS ( petrositis ) OR TITLE-ABS-KEY ( "malignant otitis externa" OR "otitis externa" ) OR INDEXTERMS ( "otitis externa" ) ) | 8090 |
| 7 | TITLE-ABS-KEY ( "diagnostic imaging" OR "tomography, emission-computed" OR "positron-emission tomography" OR "positron emission tomography computed tomography" OR tomography OR"tomography, emission-computed" OR "positron-emission tomography" OR "positron emission tomography computed tomography" ) | 2361506 |
| 8 | TITLE-ABS-KEY ( "positron*emission tomography" OR pet OR pet*ct* OR ct*pet* OR "fluorodeoxyglucose f18" OR fluorodeoxyglucose OR fludeoxyglucose OR fdg OR fdg*pet* OR flu*rodeoxyglucose ) | 304502 |
| 9 | ( TITLE-ABS-KEY ( "positron*emission tomography" OR pet OR pet*ct* OR ct*pet* OR"fluorodeoxyglucose | 2489933 |

| | | |
|---|---|---|
| | f18" OR fluorodeoxyglucose OR fludeoxyglucose OR fdg OR fdg*pet* ORflu*rodeoxyglucose ) ) OR ( TITLE-ABS-KEY ( "diagnostic imaging" OR "tomography, emission-computed"OR "positron-emission tomography" OR "positron emission tomography computed tomography" ORtomography OR "tomography, emission-computed" OR "positron-emission tomography" OR "positron emission tomography computed tomography" ) ) | |
| 10 | ( ( TITLE-ABS-KEY ( "positron*emission tomography" OR pet OR pet*ct* OR ct*pet* OR "fluorodeoxyglucose f18" OR fluorodeoxyglucose OR fludeoxyglucose OR fdg OR fdg*pet* OR flu*rodeoxyglucose ) ) OR ( TITLE-ABS-KEY ( "diagnostic imaging" OR "tomography, emission-computed" OR "positron-emission tomography"OR "positron emission tomography computed tomography" OR tomography OR "tomography, emission-computed" OR "positron-emission tomography" OR "positron emission tomography computed tomography") ) ) AND ( ( TITLE-ABS-KEY ( ( petr* or skull or crani* or invas* or necroti* or malig* ) W/2 osteomy* ) ) OR (TITLE-ABS-KEY ( ( necroti* or malig* or invas* ) W/1 otit* W/1 extern* ) ) OR ( TITLE-ABS-KEY ( otit* W/1extern* ) ) OR ( TITLE-ABS-KEY ( petrositis OR gradenigo ) ) OR ( INDEXTERMS ( petrositis ) OR TITLE-ABS-KEY( "malignant otitis externa" OR "otitis externa" ) OR INDEXTERMS ( "otitis externa" ) ) ) | 1581 |

## Web of Science

# Web of Science Search Strategy (v0.1)

# Database: Web of Science Core Collection

# Entitlements:

- WOS.IC: 1993 to 2025

- WOS.CCR: 1985 to 2025

- WOS.SCI: 1900 to 2025

- WOS.AHCI: 1975 to 2025

- WOS.BHCI: 2005 to 2025

- WOS.BSCI: 2005 to 2025

- WOS.ESCI: 2005 to 2025

- WOS.ISTP: 1990 to 2025

- WOS.SSCI: 1900 to 2025

- WOS.ISSHP: 1990 to 2025

| # | Search Query | Results |
|---|---|---|
| 1 | TS=((petr* OR skull OR crani* OR invas* OR necroti* OR malig* ) NEAR/2 osteomy*) | 989 |
| 2 | TS=((necroti* OR malig* OR invas* ) NEAR/1 otit* NEAR/1 extern* ) | 885 |
| 3 | TS=(Otit* NEAR/1 extern*) | 3375 |

| | | |
|---|---|---|
| 4 | TS=((Petrositis OR Gradenigo )) | 257 |
| 5 | #4 OR #3 OR #2 OR #1 | 4331 |
| 6 | TS=(("Diagnostic Imaging" OR "Tomography, Emission-Computed" OR "Positron-Emission Tomography" OR "Positron Emission Tomography Computed Tomography" OR Tomography OR "Tomography, Emission-Computed" OR "Positron-Emission Tomography" OR "Positron Emission Tomography Computed Tomography" )) | 848185 |
| 7 | TS=(("Positron$emission tomography" OR PET OR PET$CT* OR CT$PET* OR "Fluorodeoxyglucose F18" OR Fluorodeoxyglucose OR fludeoxyglucose OR FDG OR FDG$PET* OR Flu$rodeoxyglucose OR 2-Flu$rine-18 OR Flu$rodeoxyglucose* )) | 303668 |
| 8 | #6 OR #7 | 1047948 |
| 9 | #8 AND #5 | 420 |

# Supplementary Data Section S2: MetaBayesDTA Full Statistical Output

Below is the full input and statistical output data from MetaBayesDTA used for the meta-analysis, for purposes of reproducibility.

## MetaBayesDTA FData

This is the data from **Table 1** of the article formatted to work with the MetaBayesDTA online software tool.

| author | year | TP | FN | FP | TN | rob_PS | rob_IT | rob_RS | rob_FT | ac_PS | ac_IT | ac_RS | reference.cat | Dx or Tx.cat | SUVMax.cts | FDGValue.cat | followup.cts |
|---|---|---|---|---|---|---|---|---|---|---|---|---|---|---|---|---|---|
| Hurstel | 2024 | 4 | 0 | 0 | 13 | 2 | 3 | 1 | 2 | 1 | 1 | 1 | clinical | Response | 4.1 | SUVMax | 3 |
| Jansen | 2025 | 5 | 0 | 5 | 10 | 3 | 3 | 3 | 3 | 2 | 1 | 3 | clinical+CT/MRI/LS | Response | 4.6 | SUVMax | 6 |
| Kulkarni | 2020 | 14 | 0 | 0 | 9 | 2 | 2 | 2 | 3 | 1 | 1 | 1 | clinical | Response | 2.5 | SUVMax | 1.7 |
| Lecolier | 2025 | 4 | 1 | 0 | 35 | 2 | 3 | 2 | 2 | 1 | 1 | 1 | clinical | Response | NA | TMV | 3 |
| Stern Shavit | 2019 | 1 | 0 | 0 | 7 | 3 | 2 | 3 | 2 | 3 | 1 | 3 | clinical | Response | NA | Qualitative | 16 |
| Thanneru | 2024 | 8 | 0 | 3 | 17 | 2 | 2 | 3 | 2 | 1 | 1 | 3 | clinical+CT/MRI/LS | Response | NA | Qualitative | 20 |
| Vion | 2020 | 4 | 0 | 4 | 3 | 2 | 2 | 3 | 1 | 1 | 1 | 2 | clinical+CT/MRI/LS | Response | 2.8 | SUVMax | 12 |
| Vosbeek | 2023 | 1 | 0 | 1 | 6 | 1 | 2 | 3 | 2 | 1 | 2 | 2 | clinical | Response | NA | Qualitative | 3 |

**Data Variables and Abbreviations:** TP, True Positive; FN, False Negative; FP, False Positive; TN, True Negative. Risk of bias domains assessed using QUADAS-2: rob_PS, Risk of Bias—Patient Selection; rob_IT, Risk of Bias—Index Test; rob_RS, Risk of Bias—Reference Standard; rob_FT, Risk of Bias—Flow and Timing. Applicability concerns assessed using QUADAS-2: ac_PS, Applicability Concerns—Patient Selection; ac_IT, Applicability Concerns—Index Test; ac_RS, Applicability Concerns—Reference Standard. Additional variables: reference.cat, reference standard category (composite clinical, microbiological, imaging, or combination); Dx or Tx.cat, diagnostic accuracy at initial presentation versus treatment response monitoring; SUVMax.cts, maximum standardised uptake value (continuous variable); FDGValue.cat, $^{18}$F-FDG positivity threshold method (visual assessment, semi-quantitative SUV-based, or qualitative scoring); followup.cts, duration of clinical follow-up in months (continuous variable).

## MetaBayesDTA Full Statistical Output

Below is the output table with meta-analysis variables as output by MetaBayesDTA software with the above input data in order to generate diagnostic accuracy measures and figures and forest plots used in the article.

| PARAMETER | POSTERIOR MEDIAN | STANDARD DEVIATION | 95% POSTERIOR INTERVAL | |
|---|---|---|---|---|
| **1** | logit(sensitivity) ( μ<sub>1</sub> ) | 2.991 | 0.708 | (1.781, 4.546) |
| **2** | logit(specificity) ( μ<sub>0</sub> ) | 2.103 | 0.625 | (0.881, 3.363) |
| **3** | Sensitivity ( logit<sup>-1</sup>(μ<sub>1</sub>)) | 0.952 | 0.037 | (0.856, 0.990) |
| **4** | Specificity ( logit<sup>-1</sup>(μ<sub>0</sub>)) | 0.891 | 0.068 | (0.707, 0.967) |
| **5** | False Positive Rate (1 - Specificity) | 0.109 | 0.068 | (0.033, 0.293) |
| **6** | Diagnostic Odds Ratio | 171.994 | 342.477 | (28.616, 1148.643) |
| **7** | Likelihood Ratio +ve | 8.7 | 6.792 | (3.172, 28.367) |
| **8** | Likelihood Ratio -ve | 0.054 | 0.042 | (0.012, 0.169) |
| **9** | Between-study Correlation( ρ ) | -0.063 | 0.445 | (-0.834, 0.800) |
| **10** | Between-study SD for logit(Sensitivity) ( σ<sub>1</sub> ) | 0.589 | 0.521 | (0.042, 1.922) |
| **11** | Between-study SD for logit(Specificity) ( σ<sub>0</sub> ) | 1.472 | 0.498 | (0.605, 2.557) |
| **12** | Cutpoint parameter ( Θ ) | 1.756 | 2.78 | (-0.508, 9.287) |
| **13** | Accuracy parameter ( Λ ) | 6.3 | 4.715 | (3.877, 19.369) |
| **14** | Shape parameter ( β ) | 0.915 | 1.077 | (-0.613, 3.585) |
| **15** | SD of cutpoint parameter ( σ<sub>θ</sub> ) | 0.376 | 0.576 | (0.016, 2.115) |
| **16** | SD of accuracy parameter ( σ<sub>α</sub> ) | 1.502 | 2.306 | (0.065, 8.458) |

# Supplementary Data Section S3: Risk of Bias QUADAS-2 Signalling Questions

For purposes of reproducibility, below are the signalling questions formulated for use with the QUADAS-2 to determine risk of bias.

## Scope and intended use

Methodological quality of included studies was assessed using **QUADAS-2** (Quality Assessment of Diagnostic Accuracy Studies-2), adapted to the clinical context of **skull base osteomyelitis / malignant (necrotising) otitis externa (SBOM/MOE/NOE)** and the index test **18F-FDG PET (PET, PET/CT, PET/MRI)**. The tool was applied to each eligible dataset (diagnosis and/or treatment-response timepoint) and recorded in a format compatible with MetaBayesDTA.

## Judgement framework

For each study (or dataset/timepoint), QUADAS-2 was assessed across four **Risk of Bias** domains (D1–D4) and three **Applicability** domains (D1–D3). Each signalling question was rated **Yes / No / Unclear**. Domain-level judgements were then assigned as **Low / Some concerns / High** as described below.

## Domain-level risk-of-bias scoring rules (MetaBayesDTA-coded)

For each Risk of Bias domain:

- **Low risk of bias** if **all** signalling questions are **Yes**
- **High risk of bias** if **any** signalling question is **No**, unless the issue was explicitly judged trivial and overridden (pre-specified overrides were not used in this review unless stated)
- **Some concerns** if **no** signalling questions are **No**, but **one or more** are **Unclear**

Applicability was judged as **Low / Some concerns / High** based on concerns regarding match to the review question, as specified below.

## D1. Patient selection

**Risk of bias: signalling questions**

1. **Was a consecutive or random sample** of patients with suspected or confirmed SBOM/MOE/NOE enrolled?
2. **Was a case–control design avoided** (i.e., no artificial selection of clear disease vs clear non-disease groups)?
3. **Did the study avoid inappropriate exclusions** (e.g., excluding very severe, very mild, or PET-negative cases in a way likely to bias accuracy)?

**Risk of bias: domain judgement**

Assign domain risk of bias (Low / Some concerns / High) using the scoring rules above.

**Applicability: patient selection and setting**

**Applicability judgement (Low / Some concerns / High):**
Are there concerns that the included patients or setting do not match the review question (e.g., markedly different SBOM/MOE spectrum, ICU-only cohorts, extreme prevalence, or atypical clinical context)?

## D2. Index test (18F-FDG PET ± CT/MRI)

**Risk of bias: signalling questions**

1. **Were index test results interpreted without knowledge** of the reference standard results?
2. **If a threshold or qualitative PET criterion was used** (e.g., SUVmax cut-off, visual uptake score), was it **prespecified** (not chosen based on study data)?

**Risk of bias: domain judgement**

Assign domain risk of bias (Low / Some concerns / High) using the scoring rules above.

**Applicability: index test**

**Applicability judgement (Low / Some concerns / High):**
Are there concerns that the PET protocol/technology/interpretation does not match the review question and typical clinical practice (e.g., atypical acquisition parameters, non-standard reading rules, or materially non-representative imaging approach)?

## D3. Reference standard

**Risk of bias: signalling questions**

1. **Is the reference standard likely to correctly classify** active SBOM/MOE/NOE (e.g., appropriate composite of histology, microbiology, surgery, and/or structured clinical/imaging follow-up)?
2. **Were reference standard results interpreted without knowledge** of the index test (PET) results?

**Risk of bias: domain judgement**

Assign domain risk of bias (Low / Some concerns / High) using the scoring rules above.

**Applicability: reference standard and target condition**

**Applicability judgement (Low / Some concerns / High):**
Are there concerns that the target condition as defined by the reference standard does not match the review question (e.g., only otitis externa without skull-base involvement, overly narrow/broad disease definition, inadequate follow-up, or incorporation of PET into the disease definition)?

## D4. Flow and timing

**Risk of bias: signalling questions**

1. **Was there an appropriate interval** between the index test (PET) and reference standard (short enough at diagnosis to avoid change in disease status; appropriate minimum follow-up for treatment response)?
2. **Did all participants receive a reference standard**, regardless of PET result?
3. **Did all participants receive the same reference standard** (or a clearly prespecified hierarchy independent of PET result), avoiding differential verification?
4. **Were all participants included in the analysis** (no major unexplained exclusions after PET or reference standard)?

**Risk of bias: domain judgement**

Assign domain risk of bias (Low / Some concerns / High) using the scoring rules above.

## Data recording

For each included study/dataset, the following were recorded: (i) Yes/No/Unclear for each signalling question, (ii) domain-level Risk of Bias judgement for D1–D4, and (iii) domain-level Applicability judgement for D1–D3.
